# *miR-6743-5p*, as a direct upstream regulator of GRIM-19, enhances proliferation and suppresses apoptosis in glioma cells

**DOI:** 10.1042/BSR20171038

**Published:** 2017-12-12

**Authors:** Fang Cao, Qiang Zhang, Wei Chen, Feng Zheng, Qishan Ran, Yang He, Yang Gao, Shengtao Yao

**Affiliations:** 1 Department of Cerebrovascular Disease, The First Affiliated Hospital of Zunyi Medical College, No. 139, Dalian Avenue, Huichuan District, Zunyi 563000, Guizhou, China; 2 Department of Stroke Unit and Neurosurgery, The First People’s Hospital of Zunyi No. 98, Fenghuang North Avenue, Huichuan District, Zunyi 563000, Guizhou, China

**Keywords:** apoptosis, cell proliferation, glioma, GRIM-19, miR-6743-5p, STAT3

## Abstract

Gene associated with retinoid-interferon-induced mortality-19 (GRIM-19) has been recognized as a tumor suppressor protein, which regulates cell growth, apoptosis, and migration by signal transducer and activator of transcription 3 (STAT3) signaling pathway and non-STAT3 pathway in glioma cells. Here, we investigated the molecular mechanisms that regulated GRIM-19 expression in glioma cells. By the TargetScan algorithm, four miRNAs, hsa-*miR-17-3p*, hsa-*miR-423-5p*, hsa-*miR-3184-5p*, and hsa-*miR-6743-5p*, were identified with the potential to bind with 3′-UTR of GRIM-19. Further miRNA inhibitor transfection and luciferase assays revealed that *miR-6743-5p* was able to directly target the 3′-UTR of GRIM-19. Additionally, *miR-6743-5p* expression was inversely related with GRIM-19 expression in glioma specimens and cell lines. Moreover, the inhibition of *miR-6743-5p* caused a significant inhibition of cell proliferation and a marked promotion of cell apoptosis in glioma cells, and this phenotype was rescued by GRIM-19 knockdown. Finally, the inhibition of *miR-6743-5p* expression suppressed the phosphorylation of STAT3, and the mRNA expression of CyclinD1 and Bcl-2, two target genes of STAT3, while *miR-6743-5p* mimic had the inversed effects. Treatment with STAT3 inhibitor AG490 partially rescued the proliferation-promoting and anti-apoptosis effects of *miR-6743-5p* overexpression or GRIM-19 knockdown. Collectively, *miR-6743-5p* may act as an oncomiRNA in glioma by targetting GRIM-19 and STAT3.

## Introduction

Glioma is the most frequent type of primary malignant brain tumor, arising from the brain or spinal cord tissues [1]. According to the levels of malignancy, the World Health Organization (WHO) classification divides gliomas into grade I to grade IV [2]. Glioblastoma multiforme (GBM, grade IV) is the most malignant and frequent type, accounting for approximately 70% of all diagnosed gliomas [3]. The prognosis of glioma remains poor because of the characteristic malignant proliferation and diffuse invasion [4]. Therefore, a better understanding of the molecular mechanisms of the occurrence and development of glioma may provide new therapeutic target for glioma and improve the prognosis of this disease.

Gene associated with retinoid-interferon-induced mortality-19 (GRIM-19, also known as NDUFA13) was initially identified as a novel cell death-regulatory molecule in a breast carcinoma cell line by the antisense knockout technique [5]. Subsequent studies showed that GRIM-19 plays essential role in normal embryonic development, cell apoptosis, and cell growth [6–10]. Recent evidence has suggested that GRIM-19 may serve as a tumor suppressor protein in various human cancers. For example, GRIM-19 mutations have been detected in some thyroid carcinomas [11]. Loss of expression of GRIM-19 has been reported in renal cell carcinoma [12], cervical cancer [13], colon cancer [14], and glioma [15]. Several reports have shown that GRIM-19 can negatively regulate the activity of signal transducer and activator of transcription 3 (STAT3) and the expression of STAT3 downstream genes, thus inhibiting cell transformation [9,13–15]. It has been stated that GRIM-19 exerted inhibitory effects on cell proliferation and migration, and promotion effects on cell apoptosis of glioma cell lines.

miRNAs, small non-coding RNA molecules (approximately 20–23 nts), function in the degradation of mRNA and post-transcriptional regulation of target gene expression through binding to the 3′-UTRs of the target gene [16]. Numerous studies have revealed altered expression of miRNAs in a wide spectrum of human cancers, including glioma [17]. These researches also have established that many miRNAs can elicit oncogenic or tumor suppressor activities in various malignancies [17,18].

In the present study, the TargetScan algorithm was used to predict the miRNAs targetting GRIM-19, and four miRNAs were predicted to target the 3′-UTR of GRIM-19. The luciferase assay confirmed that one of the four identified miRNAs *miR-6743-5p* directly targetted the 3′-UTR of GRIM-19 and regulated its expression in U251 glioma cells. GRIM-19 expression level was inversely correlated to *miR-6743-5p* expression in glioma specimens and cell lines. Further cell proliferation and apoptosis assays showed that knockdown of the expression of GRIM-19 partially rescued the effects of *miR-6743-5p* inhibitor in glioma cells. Additionally, we investigated the roles of STAT3 pathway in the functions of *miR-6743-5p*/GRIM-19.

## Materials and methods

### Cell lines

The human glioma cell lines (T98G, U251, U373, U87, and SHG44) were supplied by the Shanghai Institute of Cell Biology, Chinese Academy of Sciences (Shanghai, China). T98G, U373, and U87 were grown in Eagle’s minimum essential medium (MEM; HyClone, Logan, UT, U.S.A.), while other two cells were cultured in Dulbecco’s modified Eagle’s medium (DMEM; HyClone). Both culture media were supplemented with 10% FBS (Gibco, Carlsbad, CA, U.S.A.) and antibiotics. All cell lines were incubated at 37 °C in a humidified atmosphere containing 5% CO_2._

### Tissues’ samples

The study protocol was approved by the Clinical Research Ethics Committee from the First People’s Hospital of Zunyi and the First Affiliated Hospital of Zunyi Medical College. Written informed consent was obtained from each patient. A total of 90 glioma and 15 normal brain samples were obtained from the First People’s Hospital of Zunyi and the First Affiliated Hospital of Zunyi Medical College between January 2009 and December 2015. Tissue samples were snap-frozen in liquid nitrogen and then stored at −80°C.

### Transfection of the miRNA mimic, miRNA inhibitor, and siRNA

The miRNA inhibitor of hsa-*miR-17-3p*, hsa-*miR-423-5p*, hsa-*miR-3184-5p* and hsa-*miR-6743-5p*, hsa-*miR-6743-5p* mimic as well as miRNA inhibitor scramble (anti-miR-control) and controls of the miRNA mimic scramble (miR-control) used in our study were synthesized by Genepharm Technologies (Shanghai, China). Glioma cells at approximately 70% confluence were transfected with a final concentration of 30 nM for miRNA inhibitor or 5 nM for miRNA mimic using Lipofectamine 2000 (Invitrogen, Carlsbad, CA, U.S.A.) in accordance with protocols suggested by the manufacturer. At 48 h post transfection, cells were harvested for RNA extraction and real-time PCR analysis.

### Real-time PCR

Total RNA and miRNA was extracted from tissue samples or glioma cell lines by the TRIzol reagent (Invitrogen, Carlsbad, CA, U.S.A.). Stem-loop real-time RT-PCR was carried out to analyze miRNA expression. U6 RNA was used as an miRNA internal control. Briefly, extracted RNAs were converted into cDNAs with stem-loop reverse-transcription primers with cDNA synthesis kit (Thermo Fisher Scientific, Rockford, IL, U.S.A.). Real-time PCR was then performed using Maxima SYBR Green qPCR Master Mixes (Thermo Fisher Scientific) with miRNA or U6-specific forward primer and a universal reverse primer on ABI 7300 system (Applied Biosystems, Foster City, CA, U.S.A.) as the manufacturer suggested. The primers used here are listed in Table 1.

**Table 1:** Primers for the detection of miRNAs

Target	Primer	Primer sequence
hsa-miR-17-3p	RT primer	5′- CTCAACTGGTGTCGTGGAGTCGGCAATTCAGTTGAGAGGGATTC-3′
(MIMAT0000071)	Senses primer	5′- ACACTCCAGCTGGGACTGCAGTGAAGGCAC-3′
hsa- miR-423-5p	RT primer	5′- CTCAACTGGTGTCGTGGAGTCGGCAATTCAGTTGAGAAAGTC-3′
(MIMAT0004748)	Senses primer	5′- ACACTCCAGCTGGGTGAGGGGCAGAGAGCGA -3′
hsa-3184-5p	RT primer	5′- CTCAACTGGTGTCGTGGAGTCGGCAATTCAGTTGAGAAAAGC -3′
(MIMAT0015064)	Senses primer	5′- ACACTCCAGCTGGGTGAGGGGCCTCAGACCGA -3′
hsa-miR-6743-5p	RT primer	5′- CTCAACTGGTGTCGTGGAGTCGGCAATTCAGTTGAGGGGCCA-3′
(MIMAT0027387)	Senses primer	5′- ACACTCCAGCTGGGAAGGGGCAGGGACGGG -3′
U6	RT primer	5′- AACGCTTCACGAATTTGCGT -3′
	Senses primer	5′-CTCGCTTCGGCAGCACA-3′
Universal reverse primer		5′- TGGTGTCGTGGAGTCG-3′

Real-time PCR was performed to determine the mRNA levels of interested genes. In brief, the extracted total RNA was treated with DNase I (Roche, Indianapolis, IN, U.S.A.) to remove possible genomic DNA contamination and reverse-transcribed into cDNA with cDNA synthesis kit (Thermo Fisher Scientific). GAPDH was served as an internal control for real-time PCR. The PCR primers are listed in Table 2. Target gene expression was calculated using the 2^−ΔΔ*C*^_t_ method.

**Table 2:** Primers for the detection of mRNA expression

Primer	Primer sequence	Size (bp)
GRIM-19	F: 5′- GGCCCATCGACTACAAACGG -3′	121
(NM_015965)	R: 5′- CGCTCACGGTTCCACTTCATT -3′	
CyclinD1	F: 5′- TGGAGCCCGTGAAAAAGAGC -3′	135
(NM_053056)	R: 5′- TCTCCTTCATCTTAGAGGCCAC -3′	
Bcl-2	F: 5′- GGTGGGGTCATGTGTGTGG -3′	89
(NM_000657)	R: 5′- CGGTTCAGGTACTCAGTCATCC -3′	
GAPDH	F: 5′- CACCCACTCCTCCACCTTTG -3′	110
(NM_001256799)	R: 5′- CCACCACCCTGTTGCTGTAG -3′	

### Luciferase reporter assay

The human GRIM-19 3′-UTR carrying a wild-type (WT) or mutant (MUT) binding sequence of *miR-6743-5p* was inserted into the pGL3 vector (Promega, Madison, WI, U.S.A.) to make pGL3-GRIM-19-WT or pGL3-GRIM-19-MUT, respectively. The sequence of constructs was confirmed by DNA sequencing. Glioma cells were seeded into 24-well plates and co-transfected with *miR-6743-5p* mimic or inhibitor, and pGL3-GRIM-19-WT or pGL3-GRIM-19-MUT using Lipofectamine 2000. The luciferase activity was determined 24 h after transfection using a Dual-Glo® Luciferase assay kit (Promega), normalizing to *Renilla* luciferase activity.

### Knockdown of GRIM-19 expression

To knock down the expression of GRIM-19, the oligonucleotides of shRNA targetting human GRIM-19 (shGRIM-19, 5′-CCGGCCATCGACTACAAACGGAATTCTCGAGGAATACCTCATCTTTCCTCTTTTTTTC-3′ and 5′-AATTGAAAAAAAGAGGAAAGATGAGGTATTCCTCGAGAATT CCGTTTGTAGTCGATGGGGCCT-3′) and control shRNA (shNC) were synthesized by Generay (Shanghai, China). The oligonucleotides were annealed and subcloned into AgeI/EcoRI sites of the lentiviral vector PLKO.1. Lentivirus was produced by transfecting HEK293T cells with the lentiviral construct and lentiviral packaging vectors with Lipofectamine 2000 based on the manufacturer’s instructions. At 48 h post transfection, viruses were collected and infected glioma cells in the presence of 8 μg/ml polybrene.

### Cell proliferation assay

The cell growth was determined by using a cell-counting kit (CCK)-8 (CCK-8) (Dojindo Lab, Kumamoto, Japan). Briefly, cells were seeded in 96-well plates at 5000 cells per well. Cells were transfected with indicated miRNA mimic/inhibitor, infected with shGRIM-19 or shNC lentivirus, and/or treated with 10 μM AG490 (Selleck Chemicals, Houston, TX, U.S.A.) or vehicle (DMSO) as indicated. After incubation for indicated time periods, cells were incubated with the CCK-8 reagent for 1 h and optical density (OD) values at 450 nm were determined. Triplicates were performed at each time point.

### Evaluation of apoptosis by flow cytometry

The percentages of apoptotic cells were determined by Annexin V-FITC Apoptosis Detection Kit (Beyotime, Shanghai, China). Cells were seeded in six-well plates at 300000 cells per well and treated as described above. At 48 h after treatment, cells were harvested, washed with ice-cold PBS, and double-labeled with Annexin V-FITC and PI in the dark according to the protocols suggested by the manufacturer. Cells were immediately analyzed using a flow cytometer (BD Biosciences, San Jose, CA, U.S.A.).

### Western blotting

Total protein was extracted by using RIPA lysis buffer, resolved on SDS/polyacrylamide gel, and transferred on to nitrocellulose membranes (Millipore, Bedford, U.S.A.). Following blocking with 5% skim milk, the membranes were probed with antibodies against GRIM-19, p-STAT3, STAT3 (Abcam, Cambridge, MA, U.S.A.), and GAPDH (Cell Signaling, Danvers, MA, U.S.A.). After incubation with the horseradish peroxidase-conjugated secondary antibody (Beyotime), the blots were developed with ECL system (Bio–Rad, Richmond, CA, U.S.A.).

### Statistical analysis

Statistical analyses were conducted with GraphPad Prism software Version 6.0 (San Diego, CA, U.S.A.). Student’s *t* test and ANOVA test were carried out to determine the statistical significance between two groups and amongst more than two groups, respectively. Pearson correlation analysis was applied to evaluate the relationship between GRIM-19 and *miR-6743-5p* expression. All *in vitro* experiments were done in triplicates and repeated at least three times. The criterion for statistical significance was set at *P*<0.05.

## Results

### GRIM-19 is targetted by *miR-6743-5p*

**Figure 1:**
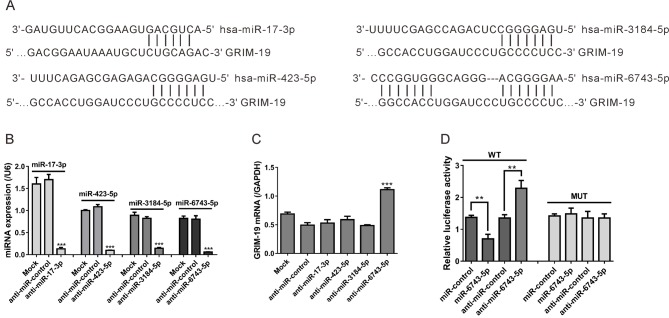
GRIM-19 is targetted by *miR-6743-5p* (**A**) The putative binding sites of the miRNAs (hsa-*miR-17-3p*, hsa-*miR-423-5p*, hsa-*miR-3184-5p*, and hsa-*miR-6743-5p*) in GRIM-19 3′-UTR as predicted by TargetScan algorithm. (**B, C**) U251 cells were transfected with various miRNA inhibitors (anti-*miR-17-3p*, anti-*miR-423-5p*, anti-*miR-3184-5p*, or anti-*miR-6743-5p*) or miRNA inhibitor scramble (anti-miR-control). Cells without any treatment were set as a negative control (Mock). At 48 h post transfection, real-time PCR analysis was performed to assess the effects of indicated miRNA inhibitor transfections on the expression of indicated miRNA (B) and *GRIM-19* mRNA (C). ***P<0.001 compared with Mock and anti-miR-control. (**D**) U251 cells were co-transfected with WT or MUT GRIM-19 3′-UTR plasmid and *anti-miR-6743-5p* mimic, miR-control, anti-*miR-17-3p*, or anti-miR-control. The relative luciferase activities were determined at 24 h post transfection, normalizing to *Renilla* luciferase activity; **P<0.01.

The TargetScan algorithm (targetscan.org) was applied to predict the miRNAs targetting GRIM-19. We found that hsa-*miR-17-3p*, hsa-*miR-423-5p*, hsa-*miR-3184-5p*, and hsa-*miR-6743-5p* had the potential to bind with GRIM-19. The predicted interaction between the miRNAs and the target sequence within the GRIM-19 3′-UTR is shown in Figure 1A.

To clarify whether these miRNAs could regulate *GRIM-19* mRNA expression in glioma, we knocked down the expression of these miRNAs in a glioma cell line, U251, and then assessed GRIM-19 expression levels. The levels of *miR-17-3p, miR-423-5p, miR-3184-5p*, and *miR-6743-5p* dropped to 7.7, 9.1, 17.6, and 7.5% of the normal levels when the cells were treated with corresponding miRNA inhibitor, respectively (Figure 1B). Negative control inhibitor (anti-miR-control) had no effects on the expression of these miRNAs. As indicated by real-time PCR analysis, the *GRIM-19* mRNA expression was significantly enhanced by the treatment of *miR-6743-5p* inhibitor (anti-*miR-6743-5p*), whereas inhibitors for other three miRNAs had no obvious effect on GRIM-19 expression (Figure 1C).

To confirm that GRIM-19 was a direct target gene of *miR-6743-5p*, we constructed GRIM-19 WT and MUT GRIM-19 3′-UTR luciferase reporter plasmids and performed the 3′-UTR luciferase assay. As shown in Figure 1D, the luciferase activity of WT GRIM-19 3′-UTR reporter was significantly reduced after the co-transfection of *miR-6743-5p* mimic in U251 cells. On the contrary, *miR-6743-5p* inhibitor (anti-*miR-6743-5p*) remarkably enhanced the luciferase activity of WT 3′-UTR reporter. The *miR-6743-5p* mimic or inhibitor had no effects on the luciferase activity of MUT 3′-UTR reporter. These data suggested that *miR-6743-5p* suppressed *GRIM-19* mRNA expression by targetting the 3′-UTR.

### Correlation analyses in glioma tissues and cell lines

**Figure 2:**
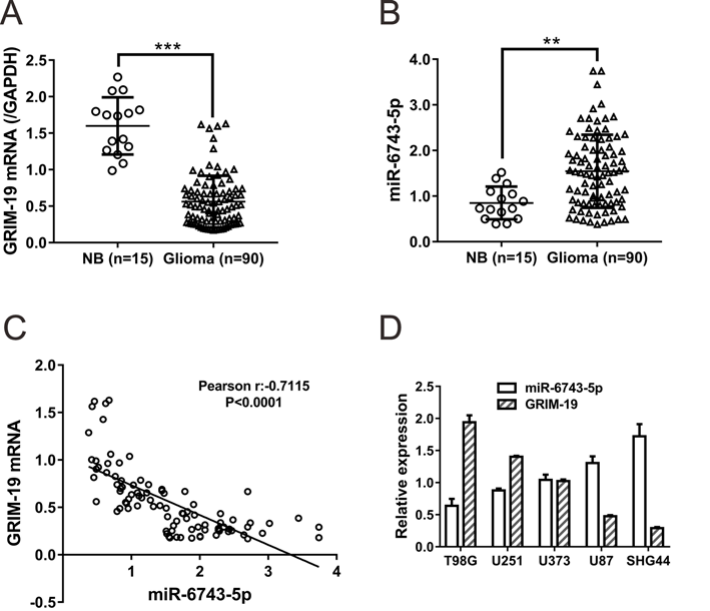
Correlation analyses in glioma tissues and cell lines (**A**,** B**) Real-time PCR analysis was performed to evaluate the expression of *miR-6743-5p* (B) and *GRIM-19* mRNA (A) in 90 glioma tissues and 15 NB. **P<0.01 and ***P<0.001. (**C**) Pearson correlation scatter plots of *miR-6743-5p* and *GRIM-19* mRNA in glioma tissues (*n*=90). (**D**) The expression of *miR-6743-5p* and *GRIM-19* mRNA were detected by real-time PCR analysis in five glioma cell lines.

The expression of *miR-6743-5p* and *GRIM-19* mRNA were assessed in 15 normal brain tissues (NB) and 90 glioma tissues by real-time PCR. Decreased *GRIM-19* mRNA expression (Figure 2A, *P*<0.001) and increased *miR-6743-5p* expression (Figure 2B, *P*<0.01) were observed in glioma tissues as compared with normal tissues.

Pearson correlation analysis revealed a statistically significant negative correlation between the expression of GRIM-19 and *miR-6743-5p* in 90 glioma tissue samples (Figure 2C). Furthermore, an inverse correlation was also in five glioma cell lines between the expression of GRIM-19 and *miR-6743-5p* (Figure 2D). These data further supported that GRIM-19 was regulated by *miR-6743-5p* in glioma.

### Opposite effects of *miR-6743-5p* and GRIM-19 on glioma cell proliferation and apoptosis

**Figure 3:**
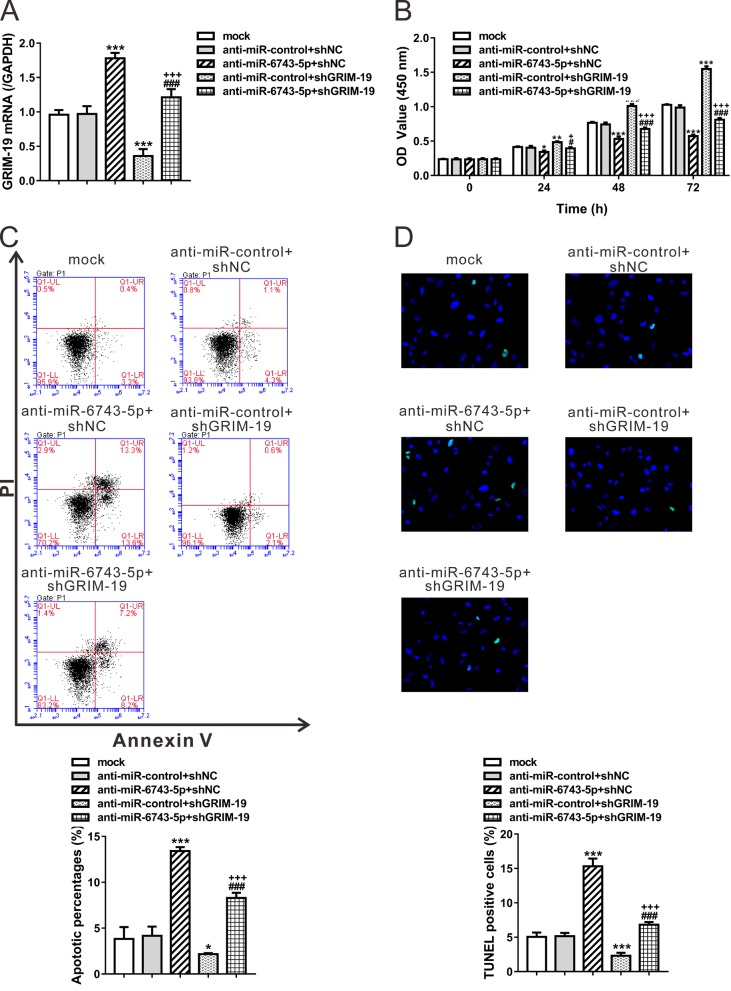
Opposite effects of *miR-6743-5p* and GRIM-19 on glioma cell proliferation and apoptosis U251 cells were transfected with anti-miR-control/anti-*miR-6743-5p*, and infected with shGRIM-19 or shNC lentivirus as indicated. Cells without any treatment were set as a negative control (Mock). (**A**) The mRNA levels of GRIM-19 were assessed at 48 h after treatment. (**B**) The proliferation curves of U251 cells at 0, 24, 48, and 72 h after treatment as determined by CCK-8 assay. (**C, D**) Cell apoptosis was determined by Annexin V/PI staining and flow cytometry analysis (**C**) and TUNEL assay (**D**) at 48 h after treatment. The representative images and the quantitative analysis are shown. The lower right quadrant (Annexin V+/PI–) represents the apoptotic cells. *P<0.05, **P<0.01, and ***P<0.001 compared with Mock and anti-miR-control + shNC; ^#^*P*<0.05 and ^###^*P*<0.001 compared with anti-*miR-6743-5p* + shNC; ^+^*P*<0.05 and ^+++^*P*<0.001 compared with anti-miR-control + shGRIM-19.

To investigate the biological effects of *miR-6743-5p*/GRIM-19 in glioma, we analyzed the proliferation and apoptosis of U251 cells after *miR-6743-5p* inhibitor treatment or GRIM-19 knockdown. GRIM-19 expression was verified by real-time PCR analyses (Figure 3A). The exposure of the *miR-6743-5p* inhibitor decreased the proliferation rate, whereas the cells with GRIM-19 knockdown showed an increased proliferation rate (Figure 3B). Similarly, the percentage of apoptotic cells was significantly higher in the cells treated with the *miR-6743-5p* inhibitor, whereas the knockdown of GRIM-19 exhibited an inverse effect as defined by Annexin V/PI staining (Figure 3C) and TUNEL staining (Supplementary Figure S1). These data indicated that *miR-6743-5p* and GRIM-19 had opposite effects on glioma cell proliferation and apoptosis. Furthermore, the cells treated with both the *miR-6743-5p* inhibitor and GRIM-19 shRNA showed significantly higher proliferation and lower apoptotic rate than the cells treated with the *miR-6743-5p* inhibitor only (Figure 3B,C, D), indicating that *miR-6743-5p* may induce cell proliferation and inhibit cell apoptosis by down-regulating GRIM-19.

### *miR-6743-5p* regulates the activity of STAT3 via GRIM-19

**Figure 4:**
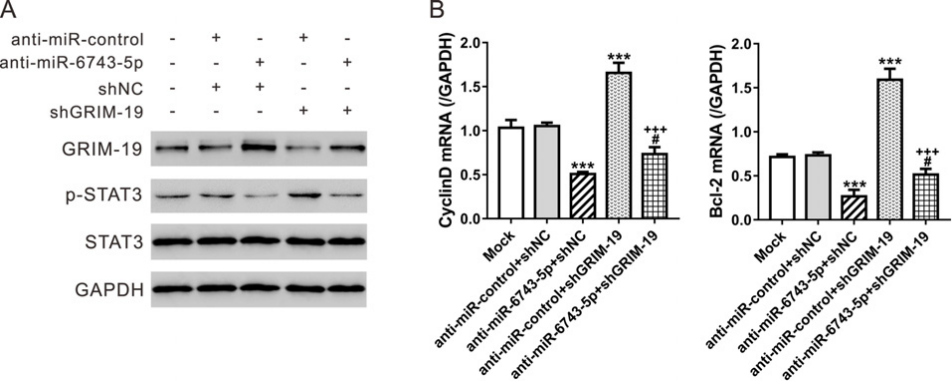
miR-6743-5p regulates the activity of STAT3 via GRIM-19 U251 cells were treated as described in Figure 3. (**A**) Protein levels of GRIM-19, p-STAT3, and STAT3 were assessed by Western blot analyses at 48 h after indicated treatment. GAPDH was detected as loading control. (**B**) The mRNA levels of CyclinD1 and Bcl-2 were detected by real-time PCR analysis at 48 h post treatment. ***P<0.001 compared with Mock and anti-miR-control + shNC; ^#^*P*<0.05 compared with anti-*miR-6743-5p* + shNC; ^+++^*P*<0.001 compared with anti-miR-control + shGRIM-19.

A previous study has shown that GRIM-19 exerted functions in glioma cells partially through STAT3-dependent pathway [15]. To explore the effect of *miR-6743-5p* on the activity of STAT3, Western blot analyses were performed in U251 cells. The *miR-6743-5p* inhibitor significantly suppressed the phosphorylation of STAT3, while the knockdown of GRIM-19 had the opposite effect (Figure 4A). Further, we analyzed the expression of STAT3 target genes, CyclinD1 and Bcl-2, at mRNA level. The mRNA expression of CyclinD1 and Bcl-2 was inhibited by the treatment of *miR-6743-5p* inhibitor, but increased by GRIM-19 knockdown (Figure 4B,C). Additionally, when compared with cells treated with the *miR-6743-5p* inhibitor, the glioma cells treated with both the *miR-6743-5p* inhibitor and shGRIM-19 exhibited higher levels of p-STAT3 as well as mRNA levels of CyclinD1 and Bcl-2 (Figure 4B), indicating that *miR-6743-5p* may active STAT3 pathway by down-regulating GRIM-19.

### *miR-6743-5p*/GRIM-19 regulates glioma cell proliferation and apoptosis via STAT3 pathway

**Figure 5:**
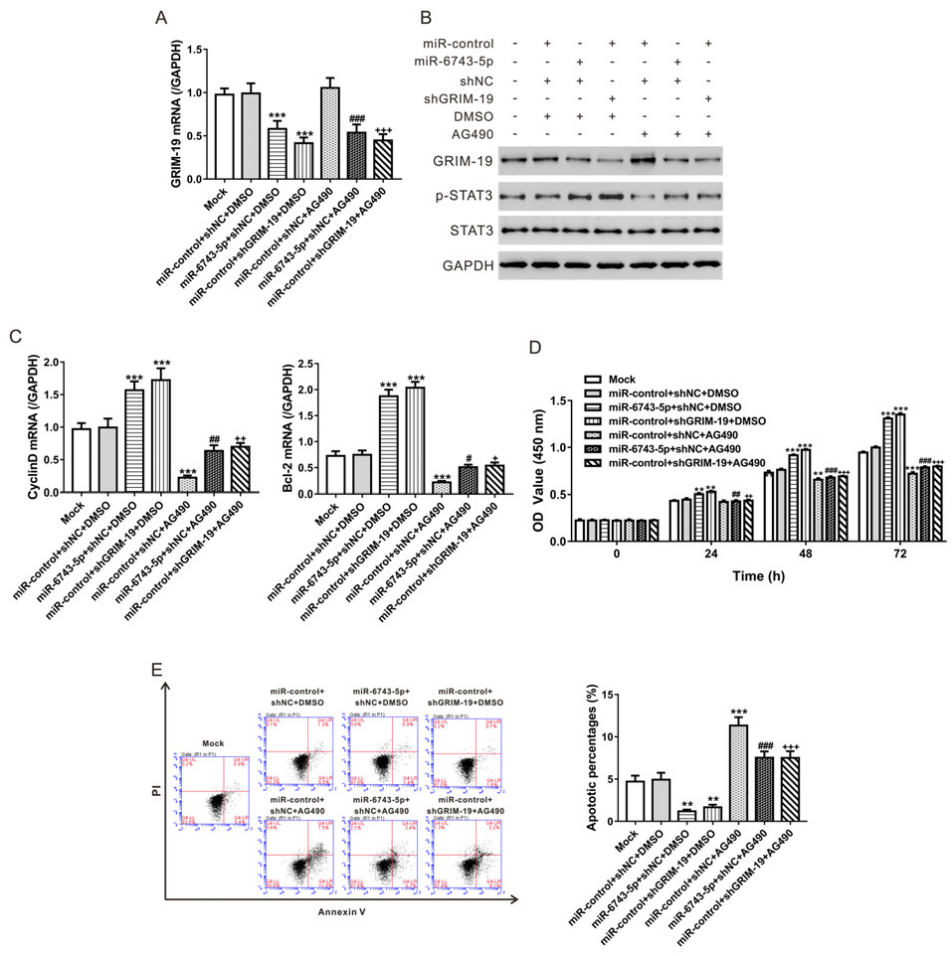
*miR-6743-5p*/GRIM-19 regulates glioma cell proliferation and apoptosis via STAT3 pathway U251 cells were transfected with *miR-6743-5p* mimic/miR-control, infected with shGRIM-19 or shNC lentivirus, and/or treated with 10 μM of AG490 or vehicle (DMSO) as indicated. Cells without any treatment were set as a negative control (Mock). (**A**) The mRNA levels of GRIM-19 at 48 h after treatment. (**B**) The protein levels of GRIM-19, p-STAT3, and STAT3 at 48 h after indicated treatment. (**C**) The mRNA levels of CyclinD1 and Bcl-2 at 48 h post treatment. (**D**) The proliferation curves as determined by CCK-8 assay. (**E**) The representative image and the quantitative analysis of apoptosis assays. **P<0.01 and ***P<0.001 compared with Mock and miR-control + shNC + DMSO; ^#^*P*<0.05, ^##^*P*<0.01, and ^###^*P*<0.001 compared with *miR-6743-5p* + shNC + DMSO; ^+^*P*<0.05, ^++^*P*<0.01, and ^+++^*P*<0.001 compared with miR-control + shGRIM-19 + DMSO.

To further confirm the involvement of STAT3 in the functions of *miR-6743-5p*/GRIM-19, a STAT3 inhibitor AG490 was used. AG490 exposure in U251 cells had no effects on the mRNA and protein expression of GRIM-19 (Figure 5A,B), but significantly decreased the p-STAT3, and the mRNA expression of CyclinD1 and Bcl-2 (Figure 5B,C). Overexpression of *miR-6743-5p* or GRIM-19 knockdown activated STAT3 pathway, which was notably suppressed by AG490 treatment (Figure 5B,C). Consistently, AG490 exposure partially rescued the proliferation-promoting and anti-apoptosis effects of *miR-6743-5p* overexpression or GRIM-19 knockdown (Figure 5D,E and Supplementary Figure S2). These data indicated that *miR-6743-5p*/GRIM-19 regulated the proliferation and apoptosis of glioma cells through regulating STAT3 activity.

## Discussion

GRIM-19 was first identified by Angell et al. [5] as a novel cell death-regulatory molecule. Since its identification, GRIM-19 is considered to actively function in embryonic development, cell growth, and apoptosis [6–10]. Besides, it may exert tumor-suppressive role in human cancers [11–14]. The present study focussed on the regulation of GRIM-19 expression in glioma. To the best of our knowledge, there are three major findings of the present study that have not been reported. First, we have shown for the first time that *miR-6743-5p* directly targetted GRIM-19 and down-regulated its expression in glioma cells. Second, the suppression of *miR-6743-5p* caused a significant inhibition of cell proliferation and a marked induction of cell apoptosis in glioma cells, and this phenotype was rescued by GRIM-19 knockdown. Last, treatment with STAT3 inhibitor AG490 partially rescued the proliferation-promoting and anti-apoptosis effects of *miR-6743-5p* overexpression or GRIM-19 knockdown.

Aberrant expression of hundreds of miRNAs has been reported in GBM tissues [17]. A number of miRNAs, such as *miR-9* [19], *miR-21* [20], *miR-125b* [21], and *miR-221/222* [22], play important roles in the progression and development of glioma via repressing the expression of tumor suppressor genes. Considering the post-transcriptional regulatory role of miRNAs, we supposed that miRNAs may be involved in GRIM-19 regulation. By bioinformatics tools, we predict several miRNAs that targetted GRIM-19. Then, by specific miRNA inhibitor transfection and luciferase assays, we demonstrated that *miR-6743-5p*, a miRNA with unknown function, directly bound to the 3′-UTR of GRIM-19, thus reducing the mRNA and protein levels of GRIM-19 (Figure 1). *miR-6743-5p* was observed to be up-regulated in human glioma tissues compared with normal brain tissues. The lower expression of GRIM-19 in glioma tissues was consistent with the previous study [15]. Moreover, an inverse correlation between the expression of *miR-6743-5p* and GRIM-19 was observed in glioma tissues and cell lines (Figure 2). Furthermore, the inhibition of *miR-6743-5p* expression suppressed the proliferation and induced apoptosis of U251 glioma cells (Figure 3), while *miR-6743-5p* mimic had the inversed effects (Figure 5). In addition, knockdown of GRIM-19 expression could reverse the effects of *miR-6743-5p* inhibitor (Figure 3), suggesting that *miR-6743-5p* exerts oncogenic function via targetting GRIM-19.

STAT3 plays a critical role in cell growth, anti-apoptosis, and cell differentiation. It is a tumor suppressor and constitutively active in glioma [23,24]. A previous study has reported that p-STAT3 expression is inversely correlated to GRIM-19 expression in gliomas and that GRIM-19 negatively regulates STAT3 activity in glioma cells [13]. Here, the inhibition of *miR-6743-5p* expression suppressed the phosphorylation of STAT3, and the mRNA expression of CyclinD1 and Bcl-2, two target genes of STAT3 (Figure 4), while *miR-6743-5p* mimic had the inversed effects (Figure 5). Furthermore, GRIM-19 knockdown could rescue the effects of *miR-6743-5p* inhibitor on STAT3 (Figure 4). The exposure of STAT3 inhibitor AG490 could partially abolish the proliferation-promoting and anti-apoptosis effects of *miR-6743-5p* overexpression or GRIM-19 knockdown (Figure 5). These findings suggested that STAT3 was involved in the functions of *miR-6743-5p*/GRIM-19 in glioma.

In conclusion, the current study explored the molecular mechanisms accounting for the dysregulation of GRIM-19 in glioma cells and identified that *miR-6743-5p* directly targetted GRIM-19. Furthermore, *miR-6743-5p* plays an essential role in the proliferation and apoptosis of glioma cells through modulating GRIM-19/STAT3.

## Supporting information

**Figure S1:** U251 cells were transfected with anti-miR-control/anti-miR-6743-5p, and infection with shGRIM-19 or shNC lentivirus as indicated. Cells without any treatment were set as a negative control (Mock). After 48 h, cells were fixed and subjected to TdT-mediated-dUTP nick end labeling (TUNEL) staining (Green) with In Situ Cell Death Detection Kit (Roche) according to the manufacturer’s protocol. Cells were counterstained with DAPI (blue). The representative images (A) and the quantitative analysis (B) of TUNEL assays are shown. ***P<0.001 vs. Mock and anti-miR-control+shNC; ###P<0.001 vs. anti-miR-6743-5p+shNC; +++P<0.001 vs. anti-miR-control+shGRIM-19.

**Figure S2:** U251 cells were transfected with miR-6743-5p mimic/miR-control, infected with shGRIM-19 or shNC lentivirus, and/or treated with 10 with 10 μM AG490 or vehicle (DMSO) as indicated. Cells without any treatment were set as a negative control (Mock). After 48 h, cells were fixed and subjected to TUNEL staining. The representative images (A) and the quantitative analysis (B) of TUNEL assays are shown. *P<0.05, **0.01 and ***0.001 vs. Mock and miR-control+shNC+DMSO; ###P<0.001 vs. miR-6743-5p+shNC+DMSO; +++P<0.001 vs. miR-control+shGRIM-19+DMSO.

## Abbreviations

CCK-8: cell-counting kit-8
GAPDH: glyceraldehyde-3-phosphate dehydrogenase
GBM: glioblastoma multiforme
GRIM-19: gene associated with retinoid-interferon-induced mortality-19
MUT: mutant
PI: propidium iodide
RIPA: radio radioimmunoprecipitation assay
RT-PCR: reverse transcription PCR
STAT3: signal transducer and activator of transcription 3
TUNEL: terminal deoxynucleotidyltransferase-mediated dUTP nick end labeling
WT: wild-type

## References

[1] Sathornsumetee S. and Rich J.N. (2006) New treatment strategies for malignant gliomas. Expert Rev. Anticancer Ther. 6, 1087–1104 16831080 10.1586/14737140.6.7.1087

[2] Louis D.N., Ohgaki H., Wiestler O.D., Cavenee W.K., Burger P.C., Jouvet A. (2007) The 2007 WHO classification of tumours of the central nervous system. Acta Neuropathol. (Berl.) 114, 97–109 17618441 10.1007/s00401-007-0243-4 PMC1929165

[3] Inda M.M., Bonavia R. and Seoane J. (2014) Glioblastoma multiforme: a look inside its heterogeneous nature. Cancers (Basel) 6, 226–239 24473088 10.3390/cancers6010226 PMC3980595

[4] Khan U.A., Bhavsar A., Asif H., Karabatsou K., Leggate J.R., Sofat A. (2015) Treatment by specialist surgical neurooncologists improves survival times for patients with malignant glioma. J. Neurosurg. 122, 297–302 25415070 10.3171/2014.10.JNS132057

[5] Angell J.E., Lindner D.J., Shapiro P.S., Hofmann E.R. and Kalvakolanu D.V. (2000) Identification of GRIM-19, a novel cell death-regulatory gene induced by the interferon-beta and retinoic acid combination, using a genetic approach. J. Biol. Chem. 275, 33416–33426 10924506 10.1074/jbc.M003929200

[6] Fearnley I.M., Carroll J., Shannon R.J., Runswick M.J., Walker J.E. and Hirst J. (2001) GRIM-19, a cell death regulatory gene product, is a subunit of bovine mitochondrial NADH: ubiquinone oxidoreductase (complex I). J. Biol. Chem. 276, 38345–38348 11522775 10.1074/jbc.C100444200

[7] Huang G., Lu H., Hao A., Ng D.C., Ponniah S., Guo K. (2004) GRIM-19, a cell death regulatory protein, is essential for assembly and function of mitochondrial complex I. Mol. Cell. Biol. 24, 8447–8456 15367666 10.1128/MCB.24.19.8447-8456.2004 PMC516758

[8] Sun P., Nallar S.C., Raha A., Kalakonda S., Velalar C.N., Reddy S.P. (2010) GRIM-19 and p16(INK4a) synergistically regulate cell cycle progression and E2F1-responsive gene expression. J. Biol. Chem. 285, 27545–27552 20522552 10.1074/jbc.M110.105767 PMC2934621

[9] Lufei C., Ma J., Huang G., Zhang T., Novotny‐Diermayr V., Ong C.T. (2003) GRIM‐19, a death‐regulatory gene product, suppresses Stat3 activity via functional interaction. EMBO J. 22, 1325–1335 12628925 10.1093/emboj/cdg135 PMC151078

[10] Zhang J., Yang J., Roy S.K., Tininini S., Hu J., Bromberg J.F. (2003) The cell death regulator GRIM-19 is an inhibitor of signal transducer and activator of transcription 3. Proc. Natl. Acad. Sci. U.S.A. 100, 9342–9347 12867595 10.1073/pnas.1633516100 PMC170920

[11] Maximo V., Botelho T., Capela J., Soares P., Lima J., Taveira A. (2005) Somatic and germline mutation in GRIM-19, a dual function gene involved in mitochondrial metabolism and cell death, is linked to mitochondrion-rich (Hurthle cell) tumours of the thyroid. Br. J. Cancer 92, 1892–1898 15841082 10.1038/sj.bjc.6602547 PMC2361763

[12] Alchanati I., Nallar S.C., Sun P., Gao L., Hu J., Stein A. (2006) A proteomic analysis reveals the loss of expression of the cell death regulatory gene GRIM-19 in human renal cell carcinomas. Oncogene 25, 7138–7147 16732315 10.1038/sj.onc.1209708

[13] Zhou Y., Li M., Wei Y., Feng D., Peng C., Weng H. (2009) Down-regulation of GRIM-19 expression is associated with hyperactivation of STAT3-induced gene expression and tumor growth in human cervical cancers. J. Interferon Cytokine Res. 29, 695–704 19642906 10.1089/jir.2009.0003 PMC2988461

[14] Gong L., Luo X., Liu S., Tao D., Gong J. and Hu J. (2007) Correlations of GRIM-19 and its target gene product STAT3 to malignancy of human colorectal carcinoma. Ai Zheng 26, 683–687 17626740

[15] Zhang Y., Hao H., Zhao S., Liu Q., Yuan Q., Ni S. (2011) Downregulation of GRIM‐19 promotes growth and migration of human glioma cells. Cancer Sci. 102, 1991–1999 21827581 10.1111/j.1349-7006.2011.02059.x

[16] Huang Y., Shen X.J., Zou Q., Wang S.P., Tang S.M. and Zhang G.Z. (2011) Biological functions of microRNAs: a review. J. Physiol. Biochem. 67, 129–139 20981514 10.1007/s13105-010-0050-6

[17] Møller H.G., Rasmussen A.P., Andersen H.H., Johnsen K.B., Henriksen M. and Duroux M. (2013) A systematic review of microRNA in glioblastoma multiforme: micro-modulators in the mesenchymal mode of migration and invasion. Mol. Neurobiol. 47, 131–144 23054677 10.1007/s12035-012-8349-7 PMC3538124

[18] Iorio M.V. and Croce C.M. (2012) MicroRNA dysregulation in cancer: diagnostics, monitoring and therapeutics. A comprehensive review. EMBO Mol. Med. 4, 143–159 22351564 10.1002/emmm.201100209 PMC3376845

[19] Schraivogel D., Weinmann L., Beier D., Tabatabai G., Eichner A., Zhu J.Y. (2011) CAMTA1 is a novel tumour suppressor regulated by miR-9/9* in glioblastoma stem cells. EMBO J. 30, 4309–4322 21857646 10.1038/emboj.2011.301 PMC3199389

[20] Zhou X., Ren Y., Moore L., Mei M., You Y., Xu P. (2010) Downregulation of miR-21 inhibits EGFR pathway and suppresses the growth of human glioblastoma cells independent of PTEN status. Lab. Invest. 90, 144–155 20048743 10.1038/labinvest.2009.126

[21] Xia H.F., He T.Z., Liu C.M., Cui Y., Song P.P., Jin X.H. (2009) MiR-125b expression affects the proliferation and apoptosis of human glioma cells by targeting Bmf. Cell. Physiol. Biochem. 23, 347–358 19471102 10.1159/000218181

[22] Zhang C., Han L., Zhang A., Yang W., Zhou X., Pu P. (2010) Global changes of mRNA expression reveals an increased activity of the interferon-induced signal transducer and activator of transcription (STAT) pathway by repression of miR-221/222 in glioblastoma U251 cells. Int. J. Oncol. 36, 1503–1512 20428775 10.3892/ijo_00000637

[23] Atkinson G.P., Nozell S.E. and Benveniste E.T. (2010) NF-κB and STAT3 signaling in glioma: targets for future therapies. Expert Rev. Neurother. 10, 575–586 20367209 10.1586/ern.10.21 PMC3886813

[24] Luwor R.B., Stylli S.S. and Kaye A.H. (2013) The role of Stat3 in glioblastoma multiforme. J. Clin. Neurosci. 20, 907–911 23688441 10.1016/j.jocn.2013.03.006

