# Supplementary material for: miR-6743-5p, as a direct upstream regulator of GRIM-19, enhances proliferation and suppresses apoptosis in glioma cells
Source: Biosci Rep. 2017 Dec 12;37(6):BSR20171038. doi: 10.1042/BSR20171038 (PMC5725612; doi:10.1042/BSR20171038)
Supplement: Supplementary file 1 [file bsr20171038_Supp1.pdf]

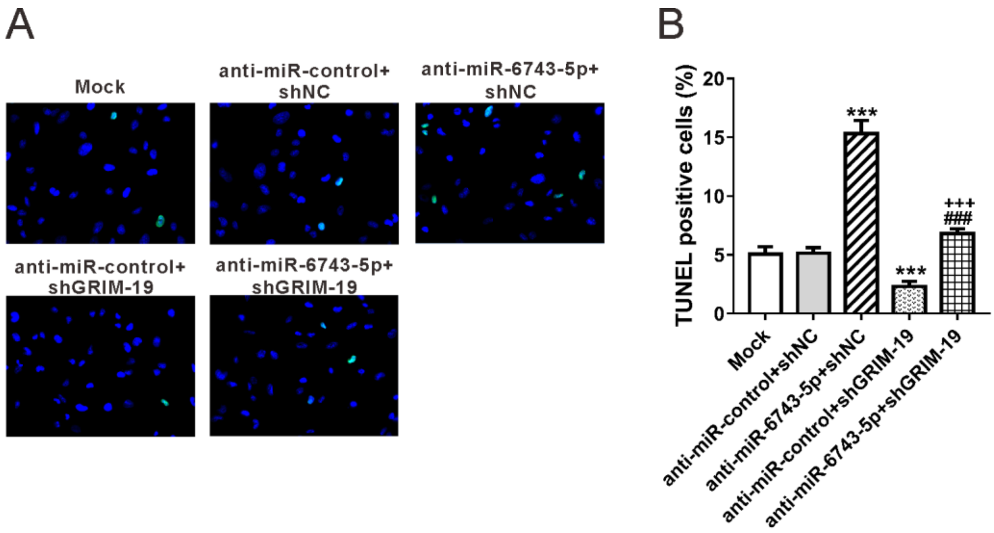

Figure S1. U251 cells were transfected with anti-miR-control /anti-miR-6743-5p, and infection with shGRIM-19 or shNC lentivirus as indicated. Cells without any treatment were set as a negative control (Mock). After 48 h, cells were fixed and subjected to TdT-mediated-dUTP nick end labeling (TUNEL) staining (Green) with In Situ Cell Death Detection Kit (Roche) according to the manufacturer's protocol. Cells were counterstained with DAPI (blue). The representative images (A) and the quantitative analysis (B) of TUNEL assays are shown. ***P<0.001 vs. Mock and anti-miR-control+shNC; ###P<0.001 vs. anti-miR-6743-5p+shNC; +++P<0.001 vs. anti-miR-control+shGRIM-19.

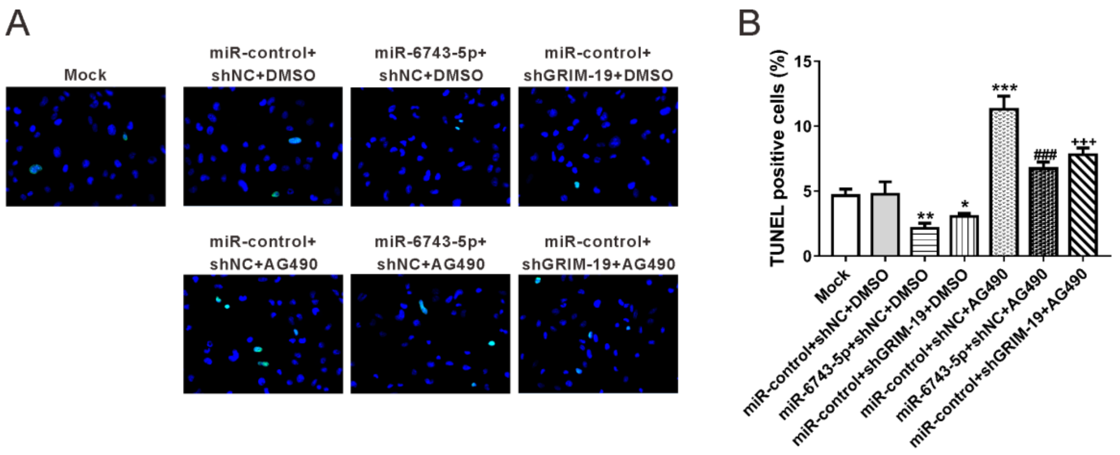

Figure S2. U251 cells were transfected with miR-6743-5p mimic/miR-control, infected with shGRIM-19 or shNC lentivirus, and/or treated with 10 μM AG490 or vehicle (DMSO) as indicated. Cells without any treatment were set as a negative control (Mock). After 48 h, cells were fixed and subjected to TUNEL staining. The representative images (A) and the quantitative analysis (B) of TUNEL assays are shown. *P<0.05, **P<0.01 and ***P<0.001 vs. Mock and miR-control+shNC+DMSO; ###P<0.001 vs. miR-6743-5p+shNC+DMSO; +++P<0.001 vs. miR-control+shGRIM-19+DMSO.
